# Editorial: Transcriptional control in normal and malignant B-lymphocytes

**DOI:** 10.3389/fimmu.2023.1185252

**Published:** 2023-03-27

**Authors:** Jerome Moreaux, Alexander A. Shtil

**Affiliations:** ^1^Institute of Human Genetics, UMR 9002 CNRS-UM, Montpellier, France; ^2^Laboratory for Monitoring Innovative Therapies, Department of Biological Hematology, CHU de Montpellier, Hôpital St. Eloi, Montpellier, France; ^3^Laboratory of Tumor Cell Death, Blokhin National Medical Research Center of Oncology, Moscow, Russia

**Keywords:** B-lymphocytes, cancer, development, gene transcription and targeted therapy, hematopoiesis

When, in 2022, *Frontiers in Immunology* offered an opportunity to launch a Research Topic, the two of us quickly agreed about its subject. One may anticipate that the key words in the title would guarantee the interest of the research community to the problems of physiological B-cell development and its disturbances in tumorigenesis. However, the endeavor exceeded our expectation. By the time of its closing in February, 2023 this Special Issue contained seven full length articles including four original studies and three comprehensive reviews. Importantly, their focus went beyond the initial title. The published works were significant contributions into the understanding of fundamental mechanisms of hematopoiesis, immunity, tumor biology and their practical applications.

Giovannini et al. raised an important issue as to how the antibody-secreting cells (ASCs) promote autoimmune disorders upon tissue infiltration. Addressing the problem of ASC heterogeneity, the authors, along with other mechanisms, analyzed the local expression of cytokines as a factor of pathological regulation by ASCs. One may suggest that this factor, as well as other characteristics of the complex ASC phenotype(s), are dependent on the involvement of specific components of the gene transcription machinery. Of special interest is transcriptional reprogramming by CDK8/19/Mediator, a mechanism of cell adaptation to a variety of extracellular cues. Advances in medicinal chemistry of small molecular weight modulators of transcriptional reprogramming may be perspective to combat autoimmune reactions.

In the review by Çakan and Gunaydin the problem of antibody diversity was dissected from the viewpoint of activation induced cytidine deaminase (AID) enzyme. Since long this enzyme has been known to control the genome integrity at various stages of B-cell differentiation. The authors performed a great work analyzing the literature and provided strong evidence in support of other physiological roles for AID, namely, epigenetic regulation, central B-cell tolerance, and humoral response. Together with the mutation burden, this enzyme becomes a broad specificity mechanism. Given its multiple functions caution is needed to consider AID as a druggable target.

Two studies analyzed multiple myeloma (MM), a B-cell malignancy characterized by the accumulation of malignant plasma cells within the bone marrow. A comprehensive review by Muylaert et al. analyzed the epigenetic dysregulation of gene expression in normal B-cell biology and in MM from the viewpoint of DNA methylation. In particular, the authors dissected new therapeutic possibilities that are about to be opened with the use of DNA methyltransferase inhibitors. Various chemotypes of these blockers, alone and/or in combinations with conventional drugs as well as with newly developing targeted antagonists, emerge as an attractive strategy.

DNA damaging drugs, a major therapeutic tool in MM, frequently fail due to activation of repair mechanisms. Ovejero et al. focused on BLM helicase, the enzyme that controls DNA integrity, as a factor of MM irresponsiveness to the alkylating agent melphalan. In patients, BLM overexpression was associated with poor outcome. In MM cell lines, targeting BLM with the small molecular weight inhibitor ML216 led to cell cycle arrest and apoptosis. Importantly, ML216 synergized with melphalan in killing wild type MM cells. In melphalan-resistant MM counterparts ML216 sensitized justifying the efficacy of this combination. Are other mechanisms of genome integrity involved in MM biology, and is BLM a therapeutic target in non-B-cell malignancies as well?

The pathogenesis of B-cell malignancies may uncover unconventional regulatory features. The study by Sha et al. established the role of pyroptosis, a specific form of cell death, in chronic lymphocytic leukemia (CLL). Three pyroptosis-related gene signatures were detected in high risk patients suggesting prognostic significance of decreased immune control. This finding paves the road for systematic investigation of individual death mechanisms in the immune cell microenvironment.

MicroRNA was the subject of the articles by Duroux-Richard et al. and Daum et al. The first study investigated plasma miRNAs in B-cell CLL patients treated with fludarabine-cyclophosphamide-rituximab (FCR) chemo-immunotherapy. Integrative omics data identified the prevalence of specific microRNA groups in responders *vs* relapse. The second work used a mouse model to demonstrate that the microRNA processing complex essential for B-cell maturation is required for T-cell dependent antibody responses. Conditional knockout of the RNA-binding protein DGCR8, a component of the above complex, prevented the maturation of the follicular zone B-lymphocytes and reduced the amounts of antigen-specific IgG-secreting cells and germinal centers.

The authors of published articles represented six countries in Europe and Asia. The Research Topic attracted attention of the readership of *Frontiers*, with hundreds-to-thousands views per each article. Therefore the initiative was a success.

We are especially grateful to the editorial team of *Frontiers*. The way of communication between the contributors, scientific editors, reviewers and technical stuff was indeed excellent. No unnecessary delays or incomplete information were encountered throughout our period of supervision. The electronic submission and revision systems were perfectly designed to operate timely and properly. Hopefully, the authors also enjoyed the process of communication with the journal.

## Author contributions

All authors listed have made a substantial, direct, and intellectual contribution to the work and approved it for publication.

